# The Importance of Teachers’ Need for Cognition in Their Use of Technology in Mathematics Instruction

**DOI:** 10.3389/fpsyg.2020.00259

**Published:** 2020-02-21

**Authors:** Lukasz Tanas, Katarzyna Winkowska-Nowak, Katarzyna Pobiega

**Affiliations:** ^1^Faculty of Psychology, SWPS University of Social Sciences and Humanities, Warsaw, Poland; ^2^Institute of Food Sciences, Warsaw University of Life Sciences, Warsaw, Poland

**Keywords:** ICT, need for cognition, teachers self-efficacy, technology acceptance, burnout, supervisor support

## Abstract

Modern technology offers an increasing number of tools for teaching mathematics, but technology adoption in schools encounters many barriers. The Technology Acceptance Model explains that technology usage is dependent on intentions, which rest on perceived ease of use and perceived usefulness. Less is known about the relationship between intentions and actual behavior. In the current study we show that the level of cognitive investment on the part of the teachers, captured by the construct of Need for Cognition (NC), is crucial in the use of technology in mathematical instruction, while controlling for a variety of background factors. Furthermore NC moderates the relationship between intentions and technology use, such that high NC weakens the relationship between the perceived usefulness of technology in pedagogy and its actual use.

## Introduction

Technology offers a set of potential tools for pedagogy. In the case of mathematics education, which has traditionally been dominated by pen and paper tasks, several broad categories of instruments are now available. Teachers can use computer algebra systems, numerical analysis software, statistical software, function graphers, and calculators, spreadsheets, geometry packages and many others. These new tools bring many new possibilities to education, but their application is often met with a variety of difficulties (Pierce and Ball, 2009).

Many of those difficulties are general and appear across different areas of technology use. The Technology Acceptance Model (TAM) (Davis, 1989; Venkatesh, 2000; Venkatesh and Davis, 2000) explains that technology use is dependent on intentions. Intentions are a result of two factors: perceived technology usefulness and perceived ease of use. As the data confirming the model show, perceived usefulness has a stronger direct impact on technology use intentions, but this might be due to the fact that perceived ease of use has both a direct and indirect effect on intentions. Directly, user friendly technology increases the intended use. Indirectly, technology which is easy to use is also perceived as more useful. Furthermore research has shown that perceived usefulness is mainly influenced by perceived job relevance of the particular technology, as well as the demonstrability and tangibility of results obtained by its use (Venkatesh and Davis, 2000). Social influence is also a factor in perceived usefulness, especially in relation to innovations. Use of innovative technology is linked to maintenance of a favorable image and status in a social group and this indirectly increases perception of its usefulness (Venkatesh and Davis, 2000). The other TAM factor influencing intentions is perceived ease of use. Ease of use is affected by the levels of general self-efficacy, lack of computer anxiety, computer playfulness as well as the degree of external support (Venkatesh, 2000). In summary, individuals who feel they have both internal control, as well as external support, enjoy dealing with technology and do not express worries about involvement in such a new complex task. They tend to perceive using technology as easy. Individuals who see concrete results and job relevance of technology, as well as feel that using it influences their status, perceive technology as useful.

Although much is already known about determinants of the intentions to use technology, much less is known about a crucial relationship between the intention to use it and actual usage of technology. Many people declare that they intend to use innovative technology, but fewer actually do (Venkatesh and Davis, 2000). In the context of teaching, using new technology requires an in important change in behavior. Often, a departure from the type of teaching that one is used to and has observed in the past. This requires an orchestration of existing pedagogical competences with the novel tools, which can be accomplished in many different ways (Drijvers et al., 2010). This process requires a substantial level of cognitive investment on the part of the teachers. On the basis of research from the field of individual differences, one can predict that people show relatively stable individual differences in the degree to which they are willing to make such investments (von Stumm and Ackerman, 2013). Cacioppo and Petty (1982) use the term Need for Cognition (NC) to describe the differences in the tendency to engage in and enjoy effortful cognitive activity.

Research shows that high levels of NC relate to an increase of cognitive resources spent specifically in response to situations placing high cognitive demands. Merely labeling a message as complex and challenging generates motivational differences in processing of this message by individuals varying in NC (See et al., 2009). High NC therefore results in high effort spent on a complex task, but can actually diminish effort in burdensome tasks, which are perceived as simple and predictable (Cacioppo and Petty, 1982; Mussel et al., 2016). High levels of NC also predict high effort when a complex task seems optional, but not personally relevant for the present moment. For tasks which are highly personally relevant or surprising NC does not moderate effort (Petty and Cacioppo, 2016; Luttrell et al., 2017).

In the context of education it has been shown that there is a significant, but rather modest positive relationship between NC and academic achievement of students, evident especially in the later grades, with a lack of such a relationship in earlier grades (Luong et al., 2017). On the other hand NC strongly predicts the tendency to seek optional education programs which allow for enriched, deep learning (Meier et al., 2014). The choice of such programs is predicted by NC, while controlling for intelligence, academic self-concept, mastery or performance goals.

There is less data on the relationship between NC and adult education, but the results match with what we observe in adolescents and young adults. Recent data shows that NC is positively related to the effectiveness of continuous education, predicting the effects of professional training for medical physicians (Hassan et al., 2015). Additionally NC mediates the relationship between age and numeracy skills. Age related decreases in numeracy can be, to a significant extent, explained by motivational factors, such as a decrease in need for cognition (Bruine de Bruin et al., 2015). These results can be summarized, by a reference to learning styles. High NC is related to engagement in deep learning strategies, critical analysis and content structuring while low NC relates to using low effort strategies such as memorizing and rehearsing (Evans et al., 2003; Cazan and Indreica, 2014).

Taking these results into account it can be argued that NC is the crucial variable responsible for the cognitive investment, which marks the difference between intention to use and actual use of technology in pedagogy. The effect of NC on technology use should be stronger in a context in which certain conditions are met: (a) when use of technology is optional, not required by the teaching curriculum; (b) when technology use is perceived as a complex and challenging task; and (c) when its adaptability and benefits from use in the context of learning are not immediately, personally visible. Stating this hypothesis in the terms of the Technology Acceptance Model: NC influences behavioral engagement in technology use to a greater extent when perceived usefulness and perceived ease of use of technology are low, rather than high. That is, NC moderates the relationship between intentions and use, in such a way that when intentions to use a particular technology in a particular context are low, the effect of NC on actual use of that technology is strong. In a case when intentions to use this technology are high, an individual is already strongly convinced of its benefits and ease of use, the effect of NC on actual technology use is weaker.

It also needs to be noted, that the direct effect of NC on technology use should be supplemented by an indirect effect. NC can be relevant for perceived ease of use of technology. Research by Venkatesh (2000) shows, for example, that computer playfulness is related to perceived ease of use of such technology. Computer playfulness is a construct defined as being specific to the use of computer technology, but it is similar to NC in that both relate to intrinsic motivation and engagement in a task “just for the sake of it.” Being intrinsically motivated to engage in a task lowers the perception of effort spent on the task, despite an objectively greater effort (Ryan and Deci, 2000). Therefore it can be argued that high NC increases the general strength of intentions to use technology, through increased perceived ease of use.

### Potential Confounders in the Relationship Between NC and Technology Use

We have argued that NC influences technology use both directly and indirectly, but there are also potential confounders which need to be taken into account when analyzing this relationship. Several variables might cause changes both in NC, as well as in technology use. The list of such contextual variables is large and a particular selection will always be subject to argument. None the less, some assumptions need to be made in order to show the relationship between NC and technology use, while holding potential confounders constant. In the current study we decided to control for (a) selected teaching practices (promotion of comprehension/pupil control); (b) general teaching self-efficacy; (c) peer and supervisor support; and (d) job burnout.

#### Promotion of Comprehension

Teachers differ in the degree to which they put emphasis on content comprehension and deep learning. It has been shown that promotion of comprehension prevents intellectual helplessness of students (Sȩdek and McIntosh, 1998). Promotion of comprehension is visible in requests of teachers for students to justify their answers, but in such a way that those requests allow for students’ individual interpretations. Therefore these justifications are not just elaborate memorizations, but actually reflect student comprehension and mistakes inherent in early phases of learning. Promotion of comprehension is therefore similar to mastery-approach learning, oriented toward developing new skills and understanding. Positive correlation between mastery goals and NC is very likely (Hoffman and Nadelson, 2010; Ranellucci et al., 2013) as well as a positive relationship between NC and deep learning (Evans et al., 2003; Cazan and Indreica, 2014). Therefore we can expect that teachers who place emphasis on deep learning, will also be likely to exert more effort in information search, as well as engage in mastery of new technological tools.

#### Student Control Ideology

Teachers have different views as to how much autonomy should be given to students in their school interactions. Autonomy can be defined as the perception of being volitional in one’s behavior (Howard et al., 2017). Autonomy does not necessarily equal independence. Rather, it is a perception of a willing choice to follow certain rules or regulations – treating them as relatively self-given. In the school context this relates to the degree to which children are given the option to influence the regulations, question teacher’s opinion and make decisions regarding course content. Autonomy is inversely related to hierarchical power structure in which the teacher is the sole controller of motivation, rewards and punishments (Howard et al., 2017). Lack of willingness to afford student autonomy is also related with higher teacher burnout (Bas, 2011). The less autonomy a teacher is willing to give the students the more likely he/she views them as irresponsible and potentially undisciplined. With high student control beliefs, order maintenance will be seen as one primary goals, and since introduction of new technology is likely to result in elevated class disturbance, teachers without autonomy preference should be less willing to engage in such behavior. Additionally, as Ryan and Deci (2000) argue, fulfillment of autonomy needs is factor in internal motivation. It is likely that teachers who provide supportive conditions for student autonomy, are themselves more likely to be characterized by internal motivation and need for cognition.

#### Teaching Self-Efficacy and Burnout

Burnout is a syndrome of interrelated feelings of emotional exhaustion, negative and detached attitude toward the people one works with and reduced feelings of personal accomplishment, as well as negative self-evaluation (Maslach et al., 2001). Teaching is generally considered as an occupation with high levels of job related psychological stress (Johnson et al., 2005) which is likely to result in burnout (Kokkinos, 2007). Self-efficacy is a personal attribute, which helps in coping with challenges (Tschannen-Moran and Hoy, 2001). Differences in self-efficacy are especially visible in responses to a novel task. For example, self-efficacy in computer use can affect perception of the ease of use before any experience with particular software or hardware (Venkatesh and Davis, 1996). Similarly, teaching self-efficacy can influence the intentions to use technology as a pedagogical tool, even without direct, hands-on experience. Teacher self-efficacy is related to teachers’ task persistence and commitment, as well as instructional style (Tschannen-Moran and Hoy, 2001). Research shows that self-efficacy and burnout explain teachers’ motivation to leave their profession. Skaalvik and Skaalvik (2016) have shown that there are two ways in which stressors affect the decision of teachers to quit their profession. Time pressure directly causes burnout and feelings of emotional exhaustion, which then predict the decision to quit. The other route is through lack of social support, especially supervisory support and trust, combined with low student motivation. This results in low-self efficacy and finally predicts the decision to quit. Taking this into account, one can expect that both burnout and teaching self-efficacy can predict general engagement in any complex and novel tasks in teachers’ daily activities.

#### Social Support

The extent to which people can count on their colleagues and supervisors in their jobs significantly affects their perception of challenges and stress (Widerszal-Bazyl and Cieślak, 2000). Perceived support from the school predicts teacher’s motivation to persist in implementation of project-based learning (Lam et al., 2010). This perception is based on feelings of collegiality as well as autonomy and competence acknowledged by the supervisors. Studies also show that social support predicts higher general control over job related challenges and this explains the negative relationship between social support and burnout (Ben-Zur and Michael, 2007). As previously mentioned, lack of supervisor support and trust is one of the main reasons for leaving the teaching profession (Skaalvik and Skaalvik, 2016). Similarly, as with self-efficacy and burnout, social support is therefore an important determinant of general job engagement and perception of challenges. Specific social support, related to particular technology (IT support) is also included in the TAM, as a factor influencing perceived ease of use (Venkatesh, 2000). In the current study we therefore aim to control for both the perception of supervisor and peer social support. In summary, in the current study we probe the relationship between NC, intentions to use technology and actual use of technology in teaching. We test for two effects. (1) That NC serves as a moderator of the relationship between intentions to use technology in teaching and actual behavior. When intentions are high, NC is not necessary for investment in behavior to take place. On the other hand, when intentions to use technology in teaching are low, NC becomes the regulator of intellectual investment; and (2) That NC is generally, positively related to intentions to use technology. We test those effects while controlling for perceived social support, self-efficacy, burnout and selected pedagogical beliefs.

## Materials and Methods

### Participants and Procedure

A total of 150 mathematics teachers (130 females, Mage = 45,15, SD = 9,5, min = 23, max = 65) from Poland took part in the study. Teachers were employed by institutions from International Standard Classification of Education (ISCED) level 1 - primary (34,7%), ISCED-2 - lower secondary (17,3%), ISCED-3 - upper secondary schools (48%). Mean teachers work experience was 19,7 years (SD = 10,03).

#### Procedure

The study was conducted in a form of an online questionnaire. Link to the study was distributed through a mailing list of an non-profit foundation, which specializes in education, as well as a publisher of mathematics textbooks in Poland. The mailing list contained about five thousand emails of teachers, mostly teachers of mathematics, who agreed to receive information from the foundation and the publisher. This mailing list was created on the basis of participation in workshops, conferences or textbook sales, related to teaching of mathematics. Data was gathered from 8.02.2018 to 27.03.2018. Questionnaires could be completed on a stationary computer or a mobile phone. There was no scale-related missing data in the study, as the questionnaire required answers to all questions. The study procedure was accepted by the Ethics Committee of the SWPS University of Social Sciences and Humanities (decision nr 31/2017).

### Measures

#### Need for Cognition – Polish, Short Version

Matusz et al. (2011) developed a 36-item scale for measurement of the construct of the Need for Cognition, with two main goals in mind: (a) to measure the universal NC construct using items in Polish which would paraphrase the original items from Cacioppo and Petty (1982); and (b) to create a scale which would include items sensitive to distinctions in a population with an elevated level of NC. The authors noted that some of the original items such as “Thinking is not my idea of fun” or “I only think as hard as I have to” are likely to show low discrimination in a population with elevated NC. Matusz et al. (2011) have shown validity, reliability of their scale, as well as its unitary structure. Unfortunately, a scale measuring a unitary construct with 36 items is not very parsimonious. Therefore, for the purpose of the current study, we created a more efficient version of this scale, similar to Cacioppo et al. (1984). We have contacted the authors and obtained raw data from the studies described in Matusz et al. (2011). Following Guadagnoli and Velicer (1988) we set the criteria which would offer a stable solution for sample size of about 100 and decided to select items with loadings above 0.5. There were 10 items that met this criterion. For full list of items see Supplementary Material. Scale includes questions such as “I like it when my life involves intellectual challenges,” answered on a 5-point scale from 1: “Definitely no” to 5: “Definitely yes.” We also ran a Confirmatory Factor Analysis (CFA) for the current data. CFA was conducted using JASP Team (2019) following Brown (2014) goodness-of-fit indices criteria. Single factor solution produced acceptable indices with SRMR = 0.049, RMSEA = 0.035, CFI = 0.983 TLI = 0.977. Residual covariance was allowed for item pair: 5–10 because of a strong conceptual overlap between these two items, both related to quitting when faced with an intellectual challenge (“I do not attempt to solve complex intellectual problems” and “I quickly give up when I cannot solve a task”). Scale reliability is good with Cronbach’s α = 0.827 (95% CI 0.782–0.865), McDonald’s ω = 0.832.

#### Teachers Student Control Ideologies

Scale was created by the Educational Research Institute (IBE, 2010) on the basis of Pupil Control Inventory (Willower et al., 1967). Scale includes 13 items and describes beliefs spanning a continuum from high to low student autonomy and hierarchical relations in the educational process e.g., “Students should not be allowed to question the opinions of teachers.” Statements are evaluated on a 5-point scale, from 1: Definitely no, to 5: Definitely yes. Because we lack current data on for the scale psychometric properties, we also CFA for this scale. Single factor model produced acceptable indices with SRMR = 0.051, RMSEA = 0.034, CFI = 0.976, TLI = 0.971. Reliability is also acceptable with α = 0.846 (95% CI 0.808–0.880), ω = 0.85.

#### Promotion of Comprehension Scale

Scale consists of nine items measuring the degree of emphasis put in pedagogy on content comprehension and deep learning e.g., “When checking what students know I require them to justify their answers” (Sȩdek, 1995). The scale stems out from studies on prevention of intellectual helplessness and teaching styles (Sȩdek and McIntosh, 1998). We lack current data on the scale psychometric properties and therefore we ran CFA. Single factor model produced acceptable indices with SRMR = 0.049, RMSEA = 0.02, CFI = 0.991, TLI = 0.987 although it must be noted that residual covariance was allowed for three item pairs: 4–9; 1–3; 2–5. There was a strong conceptual overlap between these items, which diminishes the conviction that the scale is indeed unidimensional. For example, it’s a logical necessity to “allow students to ask a question if they do not understand” (item 2) if you also declare that you “encourage students to voice out any doubts” (item 5) or in order to analyze “mistakes made during initial problem solving attempts” (item 4) it seems necessary to “allow students to communicate in their own words how they understand the concept” (item 9). The issues with these item pairs should be taken into account in any further uses of the scale and it is recommended to make proper modifications to those items in order to strengthen the evidence for scale unidimensionality. Agreement with scale items is evaluated on a 5-point scale from 1: “Definitely no” to 5: “Definitely yes.” Reliability of the scale is acceptable, with α = 0.702 (95% CI 0.624–0.768), ω = 0.709.

#### Norwegian Teachers Self-Efficacy

24-item scale measures various aspects of self-efficacy beliefs of teachers (Skaalvik and Skaalvik, 2007). Participants respond to statements starting with “Declare to what degree you are able to…” followed by various aspects of self-efficacy and a 7-point scale from 1:“I am definitely not able” to 7: “I am definitely able.” Results from the Polish adaptation of the Norwegian Teachers Self-Efficacy have shown that the structure of the scale largely differs from the original 6 factor solution and could be simplified to a 3 factor model (Baka, 2017). Because of this discrepancy between original model and the model from the Polish adaptation, for clarification an exploratory factor analysis (EFA) was performed on the current data set. CFA was not performed as it was unclear whether to test the structure of the original version or the Polish adaptation. EFA results converged on suitability of retaining a three factor solution. Three factors explained 58.9% of the variance, which is similar to results obtained by Baka (2017), but with some minor discrepancies in item factor loadings. Extracted factors were: (a) General Teaching Self-Efficacy Scale, α = 0.936 (95% CI 0.902–0.950), ω = 0.937; (b) Relationships Maintenance Self-Efficacy Scale, α = 0.741 (95% CI 0.669–0.801), ω = 0.750; (c) Discipline Maintenance Self-Efficacy Scale, α = 0.864 (95% CI 0.826–0.896), ω = 0.868. See ESM for details of this analysis.

#### Social Support

Two scales of social support were adopted from the Psychosocial Working Conditions Inventory (Widerszal-Bazyl and Cieślak, 2000). Both scales include the same eight questions, but the questions refer to either “colleagues” or “supervisors,” e.g., “To what extent you can count on your colleagues [supervisors] to help you in some concrete way?” Answers are marked on a 5-point scale from 1: Very little, to 5: Very much. Reliability of both scales is good with α = 0.958 (95% CI 0.947–0.967), ω = 0.958 for peer support and α = 0.967 (95% CI 0.958–0.974), ω = 0.967 for supervisor support.

#### Oldenburg Burnout Inventory

Inventory is a 16-item measure and includes two sub-scales: exhaustion and distancing (Halbesleben and Demerouti, 2005), with a Polish adaptation by Baka and Basińska (2016). Exhaustion is defined as feelings of intense physical, affective and cognitive strain related to job demands, e.g., “I can tolerate the pressure of my work very well.” Distancing relates to disengagement from work in general or work content; beliefs that one’s work is not interesting, challenging and satisfying and one is not willing to continue in this occupation, e.g., “Lately, I tend to think less at work and do my job almost mechanically.” Agreement with statements is evaluated on a 5-point scale from 1: “Definitely no” to 5: “Definitely yes.” Half of the items are positively and half are negatively worded. Reliability of both sub-scales is good with α = 0.816 (95% CI 0.768–0.857), ω = 0.832 for Disengagement and α = 0.862 (95% CI 0.826–0.893), ω = 0.866 for Exhaustion. Both scales are highly positively correlated, *r*(150) = 0.765, *p* < 0.001 and are summarized into one burnout score for further analyses.

#### ICT Acceptance Scale

ICT acceptance is an 8-item index based on Technology Acceptance Model (Davis, 1989; Venkatesh, 2000). As the study rationale did not require a separation of the intention to use technology, from perceived ease-of-use, as well as perceived usefulness, the scale includes items from all of those components. Usefulness was measured by items such as “Thanks to technology, I have more control over the tasks performed,” perceived ease-of-use: “Learning to use technological tools is easy.” and intention to use technology: “I will often use ICT in the future.” Statements are evaluated on a 5-point scale, from 1: Definitely no, to 5: Definitely yes. Reliability of the scale is good with α = 0.871 (95% CI 0.838–0.900), ω = 0.876. In order to provide data for the structure of the scale an exploratory factor analysis (EFA) was performed. Analysis was done using IBM SPSS Statistics 24 for Windows (IBM Corporation, 2016). Kaiser–Meyer–Olkin (KMO) measure of sampling adequacy was expected to be above.5 (Kaiser, 1974). The Bartlett’s Test of Sphericity was expected to be significant (*p* < 0.05) for factor analysis to be suitable. For the EFA results for Kaiser–Meyer–Olkin (KMO) = 0.888, Bartlett’s Test of Sphericity was significant, χ2 = 542.8, *p* < 0.001, therefore principal component analysis (PCA) was run. SPSS R-Menu v2.0 was used for determining the criteria for retaining factors in EFA (Courtney and Gordon, 2013). Velicer’s Squared Minimum Average Partial test suggested a 1 factor solution and Comparative Data test (Ruscio and Roche, 2012) suggested that moving from 1 factor to 2 factor solution did not provide statistically significant improvement to model fit (*p* = 0.164). In summary, test results converged on the suitability of retaining a 1 factor solution. This factor explains 53.7% of the variance. Cut-off value of 0.40 was used for analysis of factor loadings (Hair et al., 2013). Analysis of coefficients from the component matrix suggests that all items load to a single factor and no coefficient drops below the cut-off value.

#### Complexity of ICT Use

Complexity of the current use of technology is a self-report declaration, which is composed of four cafeteria questions. (a) What ICT tools do you currently use in teaching?; (b) What do you use ICT for?; (c) Where do you get the content and classroom scenarios from?; (d) How do you communicate with students via ICT?. Each cafeteria answer has a hidden weight, which corresponds with the complexity of the use of particular method. Weights were specified by the authors before the start of the study, on the basis of personal experience with technology use in training programs for mathematics teachers in Poland. Main criterion for assigning weights is the complexity, specificity and rarity of the particular technology use. For example sharing educational material on social networks or via e-mail is given less weight than sharing it on one’s own website or other webpages. Creating educational materials from scratch is given more weight than downloading ready-made scripts. Summary of the cafeteria options and weights are described in Table 1.

**TABLE 1:** Summary of cafeteria answers for the Complexity of ICT Use scale.

Question	Cafeteria answer options	Weights
Which ICT tools do you use in teaching?	Office (Word, Excel, Powerpoint) Libre office or similar open-source package E-learning platform Electronic journal Cloud-based software (e.g., Google, Microsoft) Communicators (e.g., Skype) E-mail [Other]	1 1 1 1 1 1 1
What do you use ICT for?	Using ICT for in class presentations For communication with students For communication with parents (e-mail, e-journal) For communication with other teacher (e,g., Bulletin boards; discussion forums) For assignment and checking of homework. For assigning additional tasks, pointing to interesting web-content. For conducting interactive tasks For sharing my didactic knowledge, e.g., creating publicly available didactic content for or with others. [Other]	1 1 1 1 2 2 2 3 1
Where do you get your content for ICT classes?	I find it online, created by other teachers From textbooks, or textbook publishers websites From knowledge portals for teachers (e,g., Scholaris) From materials obtained at teacher conferences From materials made at workshops/training sessions From personally remixed materials I create my own content from scratch [Other]	1 1 1 1 2 2 3 1
How do you distribute ICT content to your students?	Through social networks Through e-mail On schools’ webpage On an e-learning platform On my own website On an international ICT website (e.g., Geogebra.org) [Other]	1 1 2 2 3 3 1

## Results

Descriptive statistics are presented in Table 2. Main analytical goal was to verify the relationship between NC and ICT Acceptance as well as ICT Use, while controlling for other variables. Minimal *p* < 0.05 level for significance was adopted in all analyses. Sample size in this study (*N* = 150) allows for a detection of medium effects with up to 10 predictors in multiple regression (Miles and Shevlin, 2001). Full Correlation Matrix is included in the ESM. Hierarchical multiple regressions with ICT Acceptance and ICT Use as outcomes and 10 predictors were performed with JASP 0.11.1 JASP Team (2019). In each case the null model included 9 predictors (Support_Colleague, Support_Supervisor, Self_efficacy_General, Self_efficacy_Relationships, Self_efficacy_Discipline, General_ Burnout, Comprehension_Promo, Student_Control, Work_ experience_years) and NC was entered in the first step.

**TABLE 2:** Descriptive statistics.

Variable	M	SD	Skewness	Kurtosis	Min	Max
Work_experience_years	19.7	10.03	–0.11	–0.67	0.5	45
Need_Cognition	4.09	0.51	–0.4	–0.02	2.4	5
Student_Control	2.78	0.6	0.4	–0.2	1.46	4.23
Comprehension_Promo	4.38	0.39	–0.89	1.45	2.78	5
Self_efficacy_General	5.38	0.84	–0.85	0.92	2.75	7
Self_efficacy_Relationships	5.27	0.85	–1.03	2.06	2	6.8
Self_efficacy_Discipline	5.35	0.98	–1.17	1.92	1.8	7
Support_Colleague	3.67	0.85	–0.87	0.58	1.13	5
Support_Supervisor	3.31	1.01	–0.57	–0.51	1	5
General_Burnout	2.44	0.64	0.92	1.38	1.13	4.69
ICT_Acceptance	4.11	0.67	–0.72	0.51	1.5	5
ICT_Use	23.19	7.05	–0.22	–0.37	6	40

Regression with ICT Acceptance as outcome produced an non-significant null model, *F*(9,140) = 1,58, n.s. and addition of NC produced a significant final model, *F*(10,139) = 3,6, *p* < 0.001, with Adjusted *R*^2^ = 0.15. In the final model both NC (standardized beta = 0.41) and Supervisor Support (standardized beta = 0.23) were significant predictors of ICT Acceptance. Collinearity statistics were all within accepted limits of tolerance >0.2 and VIF < 4 (Hair et al., 2010).

Regression with ICT Use as outcome produced a significant model, *F*(9,140) = 2,45, *p* < 0.01, with Adjusted *R*^2^ = 0.08. Self-Efficacy in Discipline was the only significant predictor with standardized beta = 0.31. Addition of NC produced a significant change in *R*^2^ = 0.07 in the final model, *F*(10,139) = 3,68, *p* < 0.001. NC (standardized beta = 0.33) and Self-Efficacy in Discipline (standardized beta = 0.27) were significant predictors of ICT Use in the final model. Collinearity statistics were all within accepted limits, with tolerance >0.2 and VIF < 4 (Hair et al., 2010).

**FIGURE 1:**
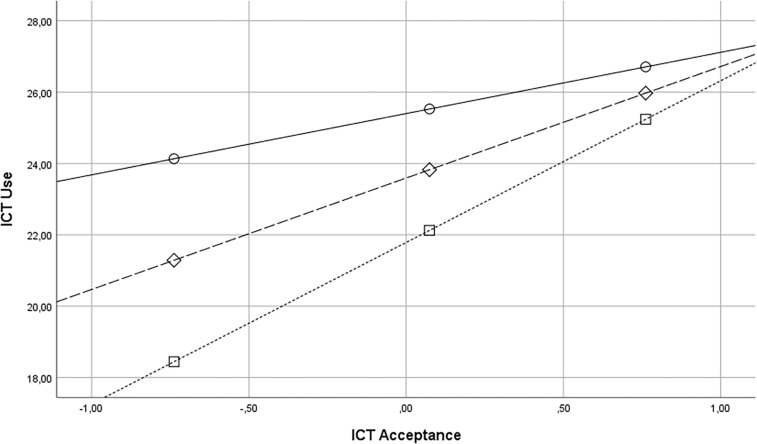
Relationship between ICT Acceptance (mean centered) and ICT Use diminishes with increasing levels of NC (mean centered). Regression lines for low NC (square, dot), *b* = 4,53, *t* = 4,79, *se* = 0,95, *p* < 0.001, medium (diamond, dash), *b* = 3,12, *t* = 3,77, *se* = 0,83, *p* < 0.001, high (circle, line), *b* = 1,72, *t* = 1,4, *se* = 1,2, *p* > 0.05.

Results so far show that NC is an important predictor of both ICT Acceptance and Complexity of ICT Use. Furthermore ICT Acceptance is moderately, positively related to Complexity of ICT Use, *r*(150) = 0.434, *p* < 0.05; in the next the step it was verified, using the PROCESS procedure created by Preacher and Hayes (2019), whether NC moderates the relationship between ICT Acceptance and ICT Use. When effect of ICT Acceptance on ICT Use is conditioned at three values of the NC: 16th (low) 50th (medium), and 84th (high) percentile, the effect becomes insignificant at the highest level of the NC (see: Figure 1), there is a significant increase in *R*^2^ = 0.02, *p* < 0.05 attributable to this moderation.

## Discussion

Results obtained in this study are in accordance with the expectations formed on the basis of previous research on NC and TAM. NC significantly predicts intentions to use ICT as well as actual ICT behavior, while controlling for burnout, self-efficacy, social support and pedagogical beliefs. Furthermore NC acts as a moderator between intentions and behavior, in such a way that the relationship between intentions and behavior is weaker, for higher levels of NC. This suggests that NC influences behavioral engagement to a greater extent when perceived usefulness and perceived ease of use of technology is low. This gives support to research showing that NC moderates effort in a context in which a task is not highly personally relevant or related to important job requirements (Petty and Cacioppo, 2016; Luttrell et al., 2017). NC becomes crucial when introducing innovative technology is not mandatory and it’s not yet clear what the job-related usefulness of the technology will be. The different pace at which changes in school curricula and official job requirements are made and at which technological advancements are made makes it almost certain that this context will be common to education. Introducing technology in teaching makes it a complex task and hard to routinize, because of constant challenges made by the software development process. On the other hand it makes teaching a challenging task, introducing novelty and incorporating the most recent ideas. It should be noted that this is vastly different from another type of change common to education - institutionally caused changes in reorganization of textbooks/learning materials, which provide a cognitive load, but do not add novelty or complexity.

It is also worth relating current results to some of the findings from the Elaboration Likelihood Model (ELM) (Cacioppo et al., 1996). According to ELM high NC leads to a stronger relationship between attitudes and behavior (Cacioppo et al., 1986, Verplanken, 1989). Generally high NC is related to deep processing of incoming information and therefore more elaborated, stronger beliefs are formed, which are then not swayed by situational factors. Greater attitude-behavior consistency is explained by the saliency of well thought of attitudes when an individual is making a decision to engage behaviorally (Pieters and Verplanken, 1995). This is contrary to the results we obtained. The relationship between attitudes and behavior was weaker for high NC individuals. This result can be explained by the finding that the strength of the intention-behavior link is different when we consider an implementation intention for a single action in a particular context or a broader goal (Sheeran, 2002). It appears that high NC is related to a broader goal of engagement in technology use, because of the complexity of this intellectual task. With low NC, there is no such general motivation and therefore what strongly predicts behavior is implementation intention based on pedagogical usefulness of technology.

Secondary results from the current study, for which we did not specify hypotheses, show that there is a lack of relationship between technology use and some important differences in teaching styles: tendency to promote comprehension and preferred degree of student autonomy. This suggests that technology *per se* does not influence these global teaching styles. Future studies could test whether it is the case that technology can both serve to decrease or increase student autonomy or be used to promote comprehension, but equally likely to promote memorization. It is likely that teachers incorporate technology into their, already established, preferred styles of interaction (Drijvers et al., 2010) and therefore a mere change in the use of technology won’t result in changes of pedagogical approach.

Other secondary results show that supervisor support, but not peer support, predicts intentions to use ICT. This result is in accordance with other studies showing that ICT supportive school leaders influence beliefs about ICT adoption in their institutions (Hatlevik and Arnseth, 2012). It confirms the TAM assumptions about the importance of norm setting in a particular environment. Perhaps surprisingly, self-efficacy beliefs of teachers were generally not related to their technology acceptance or use, apart from beliefs about efficacy in maintaining discipline, which predicted technology use. Future studies should focus on whether this can be explained by the fact that the introduction of any active pedagogical methods often involves an increase of the level of classroom noise and possible disruptions.

### Study Limitations

Before we offer some suggestions as for technology adoption in teaching, it should be noted that the current study has several limitations. Data was gathered via self-reports and on one occasion only. This suggests a potential method bias, as the measurement of intentions and declared technology use was done simultaneously. In order to avoid the confounding effect of declared intentions on retrospective of past behavior, we have tried to be as specific and concrete as possible in creating the cafeteria of answers in the Complexity of ICT Use scale. When taking into account that the questionnaire was anonymous and there was no major incentive for lying, we can assume that the self-report of actual technology use was fairly accurate.

It also needs to be mentioned that the sample might have been pre-selected on the basis of at least minimal interest in the use of modern technologies. Additionally, because the questionnaire was voluntary and not related to any governmental institution, we might have obtained a sample characterized by inflated NC in relation to the general population of teachers. Predicting this, we have used a NC scale which was especially designed to be sensitive to distinctions in a population with an elevated level of NC. We are less confident in the lack of impact of the possibly biased sample on the measures for burnout and self-efficacy. Especially for burnout, it is likely that the method of recruitment and therefore the sample, excluded teachers with high levels of this trait, which would diminish the predictive value of burnout on the variables we measured in the current study.

Additional limitation refers to the availability of the intellectual investment measures in Polish. We have used a scale which refers to NC, but there are several personality concepts which affect learning which share crucial aspects of content and definition: curiosity as a feeling of interest, curiosity as a feeling of deprivation, epistemic curiosity, typical intellectual engagement, openness to ideas and need for cognition (Litman, 2008; Mussel, 2010). These constructs share important content, but are not identical. As shown by Mussel (2013) NC is specifically related to the process of seeking and an operation of thinking. Arguably, technology use in pedagogy is also, if not more, related to the operation of learning a new skill, or creating a new artifact, as well as the process of conquering challenges (Mussel, 2013). In future studies its suggested to focus on those distinctions.

## Conclusion

Despite those limitations, we believe that the current results allow for suggestions for the potential ways to increase the use of technology in pedagogy. It seems that two routes are possible. On one hand any intervention which would increase the general level of NC would also result in an increased level of technology engagement. When thinking about such interventions it should be noted that NC is related to performance on rational thinking tasks, which are not captured by standard intelligence measures, but rather relate to heuristics, biases and critical thinking (Toplak et al., 2014). Examples of such tasks include resistance to contextual affective framing, sensitivity to base-rate information or “otherside thinking” which involves the tendency to consider both reasons consistent and inconsistent with one’s own prior beliefs. Arguably, adoption of ICT and its optimal use in education requires only an investment in technical equipment, but also an investment in the tools of the mind that practitioners in this occupation use. Most commonly education practitioners refer external barriers such as insufficient equipment, lack of software/hardware training or insufficient class time to adopt ICT in teaching (Pelgrum, 2001). However, even when equipment and training is provided, ICT adoption often gives sub-optimal results, in that it does not lead to improvements in students skills or does not bridge the gap between advantaged and disadvantaged students (OECD, 2015; Pérez-Sanagustín et al., 2017). On the other hand, for individuals with low NC, the importance of the perception of the ease of use and usefulness of technology increases as predictor of actual behavioral engagement. As shown by Gray et al. (2015) individuals with low NC might benefit especially from clearly setting a mastery goal structure in the context of technology adoption. Setting a mastery goal structure can be contrasted with setting either a performance approach or a performance avoidance structure. Unfortunately, in the context of school teachers’ performance evaluation, at least in Poland, it is more often the case that a performance avoidance structure is established. Job evaluation is aimed at avoidance of standing out negatively. This leads to enhanced risk-avoidance and challenge-avoidance, especially when perceived competence for a particular task is initially low (Harackiewicz et al., 2002) and a mixture of performance goals and being challenged in a context of low-perceived ability can produce symptoms similar to learned helplessness (Elliott and Dweck, 1988). It should also be noted that studies show that teacher training programs, which include new content, can lead to a temporary decline in teaching effectiveness (Breckwoldt et al., 2014) and time is required for experiences to accumulate which can shift this (Thomas et al., 1996). It is likely that NC can protect against some of the effects of a performance avoidance structure in that it shifts attention away from comparison with others to analysis of own performance, seeing evidence for improvement as well as seeking feedback (Luong et al., 2017). Other studies show that it is crucial the teachers are involved in active development of the learning materials and not only in the enactment of ready-made tasks (Coenders and Terlouw, 2015).

We believe that this study points to the importance of focusing on the typical intellectual investment, or need of cognition of teachers in both recruitment and training. This is likely to result in an increase of the use of ICT dependent active teaching methods in the teaching of mathematics. Active learning methods, such as peer instruction, think-pair-share or minute papers can be introduced without the use of technology (McConnell et al., 2017), but then they rarely answer to the issue raised by Bloom (1984) described as the 2 sigma problem. The problem refers to a large discrepancy in teaching effectiveness between individual tutoring and classic large scale formal education. Technology is seen as a possible vehicle for simulating some effects of individual tutoring while keeping it affordable for public education.

## Data Availability Statement

The datasets generated for this study are available on request to the corresponding author.

## Ethics Statement

The studies involving human participants were reviewed and approved by SWPS University of Social Sciences and Humanities Ethical Committee permission number 31/2017. The patients/participants provided their written informed consent to participate in this study.

## Author Contributions

LT, KW-N, and KP contributed to the design, implementation of the research and to the writing of the manuscript. LT performed the analysis of the data.

## Conflict of Interest

The authors declare that the research was conducted in the absence of any commercial or financial relationships that could be construed as a potential conflict of interest.
