# Supplementary material for: The Importance of Teachers’ Need for Cognition in Their Use of Technology in Mathematics Instruction
Source: Front Psychol. 2020 Feb 21;11:259. doi: 10.3389/fpsyg.2020.00259 (PMC7046624; doi:10.3389/fpsyg.2020.00259)
Supplement: Supplementary file 1 [file Data_Sheet_1.docx]

**Electronic Supplementary Material (ESM)**

## Correlation Matrix

| **Pearson Correlations** | | | | | | | | | | | | | |
| --- | --- | --- | --- | --- | --- | --- | --- | --- | --- | --- | --- | --- | --- |
|  | |  | |  | | **Pearson's r** | | **p** | | **Lower 95% CI** | | **Upper 95% CI** | |
| Work_experience_years |  | - |  | Self_efficacy_General |  | 0.092 |  | 0.263 |  | -0.069 |  | 0.249 |  |
| Work_experience_years |  | - |  | Self_efficacy_Relationships |  | 0.176 |  | 0.031 |  | 0.016 |  | 0.327 |  |
| Work_experience_years |  | - |  | Self_efficacy_Discipline |  | 0.189 |  | 0.021 |  | 0.029 |  | 0.339 |  |
| Work_experience_years |  | - |  | Student_Control |  | -0.174 |  | 0.034 |  | -0.325 |  | -0.014 |  |
| Work_experience_years |  | - |  | Promotion_Comprehention |  | 0.099 |  | 0.227 |  | -0.062 |  | 0.255 |  |
| Work_experience_years |  | - |  | Support_Colleague |  | -0.066 |  | 0.422 |  | -0.224 |  | 0.095 |  |
| Work_experience_years |  | - |  | Support_Supervisor |  | 0.016 |  | 0.845 |  | -0.145 |  | 0.176 |  |
| Work_experience_years |  | - |  | Burnout_Distance |  | -0.134 |  | 0.102 |  | -0.288 |  | 0.027 |  |
| Work_experience_years |  | - |  | Burnout_Exhaust |  | 0.009 |  | 0.915 |  | -0.152 |  | 0.169 |  |
| Work_experience_years |  | - |  | Need_Cognition |  | 0.152 |  | 0.063 |  | -0.008 |  | 0.305 |  |
| Work_experience_years |  | - |  | ICT Use |  | 0.157 |  | 0.055 |  | -0.003 |  | 0.310 |  |
| Work_experience_years |  | - |  | ICT Acceptance |  | 0.161 |  | 0.049 |  | 4.800e -4 |  | 0.313 |  |
| Work_experience_years |  | - |  | Age |  | 0.911 |  | < .001 |  | 0.879 |  | 0.935 |  |
| Self_efficacy_General |  | - |  | Self_efficacy_Relationships |  | 0.626 |  | < .001 |  | 0.517 |  | 0.714 |  |
| Self_efficacy_General |  | - |  | Self_efficacy_Discipline |  | 0.722 |  | < .001 |  | 0.636 |  | 0.791 |  |
| Self_efficacy_General |  | - |  | Student_Control |  | -0.159 |  | 0.052 |  | -0.312 |  | 0.001 |  |
| Self_efficacy_General |  | - |  | Promotion_Comprehention |  | 0.287 |  | < .001 |  | 0.133 |  | 0.428 |  |
| Self_efficacy_General |  | - |  | Support_Colleague |  | 0.305 |  | < .001 |  | 0.152 |  | 0.443 |  |
| Self_efficacy_General |  | - |  | Support_Supervisor |  | 0.281 |  | < .001 |  | 0.127 |  | 0.423 |  |
| Self_efficacy_General |  | - |  | Burnout_Distance |  | -0.450 |  | < .001 |  | -0.570 |  | -0.313 |  |
| Self_efficacy_General |  | - |  | Burnout_Exhaust |  | -0.432 |  | < .001 |  | -0.554 |  | -0.292 |  |
| Self_efficacy_General |  | - |  | Need_Cognition |  | 0.465 |  | < .001 |  | 0.329 |  | 0.582 |  |
| Self_efficacy_General |  | - |  | ICT Use |  | 0.219 |  | 0.007 |  | 0.060 |  | 0.366 |  |
| Self_efficacy_General |  | - |  | ICT Acceptance |  | 0.127 |  | 0.122 |  | -0.034 |  | 0.281 |  |
| Self_efficacy_General |  | - |  | Age |  | 0.054 |  | 0.509 |  | -0.107 |  | 0.213 |  |
| Self_efficacy_Relationships |  | - |  | Self_efficacy_Discipline |  | 0.621 |  | < .001 |  | 0.511 |  | 0.710 |  |
| Self_efficacy_Relationships |  | - |  | Student_Control |  | -0.099 |  | 0.227 |  | -0.255 |  | 0.062 |  |
| Self_efficacy_Relationships |  | - |  | Promotion_Comprehention |  | 0.296 |  | < .001 |  | 0.143 |  | 0.436 |  |
| Self_efficacy_Relationships |  | - |  | Support_Colleague |  | 0.363 |  | < .001 |  | 0.215 |  | 0.495 |  |
| Self_efficacy_Relationships |  | - |  | Support_Supervisor |  | 0.305 |  | < .001 |  | 0.152 |  | 0.443 |  |
| Self_efficacy_Relationships |  | - |  | Burnout_Distance |  | -0.347 |  | < .001 |  | -0.481 |  | -0.198 |  |
| Self_efficacy_Relationships |  | - |  | Burnout_Exhaust |  | -0.280 |  | < .001 |  | -0.421 |  | -0.125 |  |
| Self_efficacy_Relationships |  | - |  | Need_Cognition |  | 0.267 |  | < .001 |  | 0.112 |  | 0.410 |  |
| Self_efficacy_Relationships |  | - |  | ICT Use |  | 0.116 |  | 0.156 |  | -0.045 |  | 0.272 |  |
| Self_efficacy_Relationships |  | - |  | ICT Acceptance |  | 0.173 |  | 0.035 |  | 0.013 |  | 0.324 |  |
| Self_efficacy_Relationships |  | - |  | Age |  | 0.161 |  | 0.050 |  | 3.621e -4 |  | 0.313 |  |
| Self_efficacy_Discipline |  | - |  | Student_Control |  | -0.222 |  | 0.006 |  | -0.369 |  | -0.064 |  |
| Self_efficacy_Discipline |  | - |  | Promotion_Comprehention |  | 0.357 |  | < .001 |  | 0.208 |  | 0.489 |  |
| Self_efficacy_Discipline |  | - |  | Support_Colleague |  | 0.300 |  | < .001 |  | 0.147 |  | 0.439 |  |
| Self_efficacy_Discipline |  | - |  | Support_Supervisor |  | 0.260 |  | 0.001 |  | 0.104 |  | 0.403 |  |
| Self_efficacy_Discipline |  | - |  | Burnout_Distance |  | -0.390 |  | < .001 |  | -0.518 |  | -0.245 |  |
| Self_efficacy_Discipline |  | - |  | Burnout_Exhaust |  | -0.344 |  | < .001 |  | -0.478 |  | -0.194 |  |
| Self_efficacy_Discipline |  | - |  | Need_Cognition |  | 0.428 |  | < .001 |  | 0.287 |  | 0.550 |  |
| Self_efficacy_Discipline |  | - |  | ICT Use |  | 0.309 |  | < .001 |  | 0.156 |  | 0.447 |  |
| Self_efficacy_Discipline |  | - |  | ICT Acceptance |  | 0.223 |  | 0.006 |  | 0.065 |  | 0.370 |  |
| Self_efficacy_Discipline |  | - |  | Age |  | 0.122 |  | 0.138 |  | -0.039 |  | 0.277 |  |
| Student_Control |  | - |  | Promotion_Comprehention |  | -0.147 |  | 0.073 |  | -0.300 |  | 0.014 |  |
| Student_Control |  | - |  | Support_Colleague |  | -0.287 |  | < .001 |  | -0.428 |  | -0.133 |  |
| Student_Control |  | - |  | Support_Supervisor |  | -0.178 |  | 0.029 |  | -0.329 |  | -0.018 |  |
| Student_Control |  | - |  | Burnout_Distance |  | 0.340 |  | < .001 |  | 0.190 |  | 0.475 |  |
| Student_Control |  | - |  | Burnout_Exhaust |  | 0.364 |  | < .001 |  | 0.216 |  | 0.495 |  |
| Student_Control |  | - |  | Need_Cognition |  | -0.226 |  | 0.005 |  | -0.373 |  | -0.068 |  |
| Student_Control |  | - |  | ICT Use |  | -0.105 |  | 0.200 |  | -0.261 |  | 0.056 |  |
| Student_Control |  | - |  | ICT Acceptance |  | -0.112 |  | 0.174 |  | -0.267 |  | 0.050 |  |
| Student_Control |  | - |  | Age |  | -0.191 |  | 0.019 |  | -0.341 |  | -0.032 |  |
| Promotion_Comprehention |  | - |  | Support_Colleague |  | 0.254 |  | 0.002 |  | 0.097 |  | 0.398 |  |
| Promotion_Comprehention |  | - |  | Support_Supervisor |  | 0.147 |  | 0.072 |  | -0.013 |  | 0.300 |  |
| Promotion_Comprehention |  | - |  | Burnout_Distance |  | -0.119 |  | 0.148 |  | -0.274 |  | 0.042 |  |
| Promotion_Comprehention |  | - |  | Burnout_Exhaust |  | -0.055 |  | 0.503 |  | -0.213 |  | 0.106 |  |
| Promotion_Comprehention |  | - |  | Need_Cognition |  | 0.307 |  | < .001 |  | 0.154 |  | 0.445 |  |
| Promotion_Comprehention |  | - |  | ICT Use |  | 0.183 |  | 0.025 |  | 0.024 |  | 0.334 |  |
| Promotion_Comprehention |  | - |  | ICT Acceptance |  | 0.076 |  | 0.354 |  | -0.085 |  | 0.234 |  |
| Promotion_Comprehention |  | - |  | Age |  | 0.076 |  | 0.356 |  | -0.085 |  | 0.233 |  |
| Support_Colleague |  | - |  | Support_Supervisor |  | 0.619 |  | < .001 |  | 0.509 |  | 0.709 |  |
| Support_Colleague |  | - |  | Burnout_Distance |  | -0.306 |  | < .001 |  | -0.445 |  | -0.154 |  |
| Support_Colleague |  | - |  | Burnout_Exhaust |  | -0.355 |  | < .001 |  | -0.487 |  | -0.206 |  |
| Support_Colleague |  | - |  | Need_Cognition |  | 0.114 |  | 0.163 |  | -0.047 |  | 0.270 |  |
| Support_Colleague |  | - |  | ICT Use |  | -0.002 |  | 0.984 |  | -0.162 |  | 0.159 |  |
| Support_Colleague |  | - |  | ICT Acceptance |  | 0.055 |  | 0.502 |  | -0.106 |  | 0.214 |  |
| Support_Colleague |  | - |  | Age |  | -0.076 |  | 0.355 |  | -0.234 |  | 0.085 |  |
| Support_Supervisor |  | - |  | Burnout_Distance |  | -0.435 |  | < .001 |  | -0.556 |  | -0.295 |  |
| Support_Supervisor |  | - |  | Burnout_Exhaust |  | -0.499 |  | < .001 |  | -0.610 |  | -0.368 |  |
| Support_Supervisor |  | - |  | Need_Cognition |  | 0.133 |  | 0.105 |  | -0.028 |  | 0.287 |  |
| Support_Supervisor |  | - |  | ICT Use |  | 0.069 |  | 0.399 |  | -0.092 |  | 0.227 |  |
| Support_Supervisor |  | - |  | ICT Acceptance |  | 0.162 |  | 0.048 |  | 0.002 |  | 0.314 |  |
| Support_Supervisor |  | - |  | Age |  | 0.016 |  | 0.848 |  | -0.145 |  | 0.176 |  |
| Burnout_Distance |  | - |  | Burnout_Exhaust |  | 0.765 |  | < .001 |  | 0.689 |  | 0.824 |  |
| Burnout_Distance |  | - |  | Need_Cognition |  | -0.407 |  | < .001 |  | -0.533 |  | -0.264 |  |
| Burnout_Distance |  | - |  | ICT Use |  | -0.125 |  | 0.129 |  | -0.279 |  | 0.036 |  |
| Burnout_Distance |  | - |  | ICT Acceptance |  | -0.121 |  | 0.139 |  | -0.276 |  | 0.040 |  |
| Burnout_Distance |  | - |  | Age |  | -0.144 |  | 0.079 |  | -0.297 |  | 0.017 |  |
| Burnout_Exhaust |  | - |  | Need_Cognition |  | -0.338 |  | < .001 |  | -0.472 |  | -0.188 |  |
| Burnout_Exhaust |  | - |  | ICT Use |  | -0.085 |  | 0.300 |  | -0.242 |  | 0.076 |  |
| Burnout_Exhaust |  | - |  | ICT Acceptance |  | -0.078 |  | 0.343 |  | -0.235 |  | 0.083 |  |
| Burnout_Exhaust |  | - |  | Age |  | -0.034 |  | 0.679 |  | -0.193 |  | 0.127 |  |
| Need_Cognition |  | - |  | ICT Use |  | 0.391 |  | < .001 |  | 0.246 |  | 0.519 |  |
| Need_Cognition |  | - |  | ICT Acceptance |  | 0.367 |  | < .001 |  | 0.220 |  | 0.498 |  |
| Need_Cognition |  | - |  | Age |  | 0.123 |  | 0.133 |  | -0.038 |  | 0.278 |  |
| ICT Use |  | - |  | ICT Acceptance |  | 0.434 |  | < .001 |  | 0.294 |  | 0.556 |  |
| ICT Use |  | - |  | Age |  | 0.135 |  | 0.099 |  | -0.026 |  | 0.289 |  |
| ICT Acceptance |  | - |  | Age |  | 0.158 |  | 0.053 |  | -0.002 |  | 0.311 |  |
|  | | | | | | | | | | | | | |

**Materials and measures**

***Need for Cognition Scale - Short Version.*** Scale includes 10 items from the Need for Cognition Scale (Matusz, Traczyk, & Gąsiorowska, 2011). Asterix (*) marks reverse-scored items.

Items:

1 Mogę spędzić wiele godzin na rozważaniach nad jakimś intelektualnym problemem. / I can spend hours thinking about an intellectual problem.

2 Wolę nauczyć się, jak rozwiązać problem, niż dostać gotowe rozwiązanie. / I prefer to learn how to solve a problem than to get a ready solution.

3 Wolę zadania, które wymagają ode mnie całkowitej koncentracji, niż te, których rozwiązanie przychodzi mi bez trudu. / I prefer tasks that require total concentration from me, than those whose solution comes to me without difficulty.

4 *Staram się wybierać zadania, które są mało skomplikowane. / * I try to choose tasks that are not very complicated.

5 *Szybko się poddaję, gdy nie mogę rozwiązać jakiegoś zadania./ * I quickly give up when I cannot solve a task.

6 Poszukuję wielu nowych rozwiązań tego samego problemu. / I am looking for many new solutions to the same problem.

7 Lubię, gdy życie stawia przede mną intelektualne wyzwania. / I like it when life gives me intellectual challenges.

8 Poszerzanie wiedzy na jakiś temat sprawia mi przyjemność. / It makes me happy to expand my knowledge about a subject.

9 Zwykle zgłębiam problem, nawet, gdy inni są już zadowoleni z rozwiązania. / I usually explore the problem, even when others are satisfied with the solution.

10 *Nie podejmuję się rozwiązywania złożonych problemów intelektualnych. / * I do not attempt to solve complex intellectual problems.

***Promotion of Comprehension Scale*** *[Skala Promowania Rozumienia]* (Sędek, 1995)

Items:

1 Uczulam na te miejsca w nowym materiale, gdzie łatwo o pomyłkę, błąd w interpretacji, itp. / I point students attention to these places in the new material, where it is easy to make a mistake, or misinterpret them.

2 Pozwalam na to, żeby uczniowie przerywali mi wykładanie i zadawali pytanie, jeżeli czegoś nie rozumieją. / I allow students to interrupt my lecture and ask a question if they do not understand something.

3 Kiedy wprowadzam nowe pojęcia, dokładnie je tłumaczę. / When I introduce new concepts, I explain them exactly.

4 Wspólnie z uczniami analizujemy błędy, popełniane przy początkowych próbach opanowania nowego materiału. / Together with students, we analyze mistakes made during initial attempts to learn new material.

5 Po zakończeniu przedstawiania nowego materiału zachęcam uczniów do zgłaszania wątpliwości i problemów. / After completing the presentation of the new material, I encourage students to voice out any doubts and problems.

6 Przy sprawdzaniu wiedzy uczniów wymagam przeprowadzenia rozumowania, uzasadniania pewnej tezy, itp./ When checking students' knowledge, I require them to reason and provide justification of their argument, etc.

7 Przy odpytywaniu wymagam od ucznia samodzielnego myślenia. / When asking questions, I require independent thinking from the students.

8 Zadaję takie pytania, że trzeba się zastanowić zanim się na nie odpowie. / I ask such questions that you have to think about it before you answer it.

9 Proszę, aby uczeń przekazał własnymi słowami, co rozumie przez dane pojęcie lub określenie. / I let the students communicate in their own words what they understand by a certain concept or a term.

***Teachers Student Control Ideologies***

Items:

1 Regularne sprawdzanie wiedzy jest głównym źródłem motywacji do nauki dla uczniów. / Regular checking of knowledge is the main source of motivation for learning for students

2 Uczniom nie powinno pozwalać się na podważanie opinii nauczyciela. / Students should not be allowed to question the teacher's opinion

3 Przyjazna postawa nauczyciela wobec uczniów często prowadzi do spoufalania się z ich strony. / Friendly teacher attitude towards students often leads to lack of boundaries

4 Ważniejsze jest, aby uczniowie przestrzegali obowiązujących zasad, niż tworzyli nowe. / It is more important that students abide by existing rules than to involve them in reating new ones.

5 Jeżeli pozwoli się uczniom wychodzić do toalety bez pytania, będą tego nadużywać. / If students are allowed to go to the toilet without asking, they will abuse it.

6 Uczniom trzeba często przypominać, że ich pozycja w szkole różni się od pozycji nauczyciela. / Pupils often need to be reminded that their position at school differs from the position of the teacher

7 Należy surowo karać uczniów niszczących pomoce naukowe lub wyposażenie szkoły. / It is necessary to severely punish students who are destroying teaching aids or school equipment

8 Uczniowie nie potrafią dostrzec różnicy między demokracją i anarchią w klasie. / Students often cannot see the difference between democracy and anarchy in class

9 Uczniowie często zachowują się niewłaściwie tylko po to, by postawić nauczyciela w złym świetle. / Students often behave badly just to put the teacher in a bad light

10 Zbyt często diagnozuje się problemy z nauką u uczniów. / Too often students are excused by a diagnosis of some learning disability.

11 Poważnym błędem jest pytanie uczniów o to, jaką aktywność preferują w danej chwili./ It is a serious mistake to ask students what activity they prefer at the moment

12 Przyzwolenie na swobodne wyrażanie swoich opinii zwykle niczemu dobremu nie służy. / Permission to express your opinion freely is usually not good for anything

13 Tylko uczniom naprawdę zdolnym można pozostawić więcej swobody. / Only the best students can be given more freedom.

***ICT Acceptance Scale***

Items:

1 Dzięki narzędziom informatycznym przyjemniej wykonuję moją pracę nauczyciela. / Thanks to ICT tools, it is more pleasant to do my teacher's job.

2 Dzięki technologii mam większą kontrolę nad wykonywanymi zadaniami. / Thanks to technology, I have more control over the tasks performed.

3 Używanie technologii pozwala mi kończyć zadania szybciej. / Using technology allows me to finish tasks faster.

4 *Technologie informatyczne są często niewygodne w użyciu. / * ICT technologies are often uncomfortable to use.

5 Nauczenie się obsługi narzędzi technologicznych jest łatwe. / Learning to use technological tools is easy.

6 *Pomysł aby używać technologii informatycznych na lekcjach jest tragiczny. / * The idea to use information technology in lessons is tragic.

7 Zdecydowanie polecam innym nauczycielom stosowanie technologii informatycznych podczas lekcji. / I will definitely recommend to other teachers to use ICT during classes.

8 Będę często używać technologii informatycznych w przyszłości. / I will often use ICT in the future.

***Norwegian Teachers Self-Efficacy Scale****.*

Polish adaptation study by Baka (2017) showed a 3 factor solution. In the current study for the 24 items of the Norwegian Teachers Self-Efficacy Scale an exploratory factor analysis (EFA) was performed. Analysis was done using IBM SPSS Statistics 24 for Windows (IBM, 2016). Kaiser–Meyer–Olkin (KMO) measure of sampling adequacy was expected to be above 0.5 (Kaiser 1974). The Bartlett's Test of Sphericity was expected to be significant (p<.05) for factor analysis to be suitable. For the EFA results for Kaiser–Meyer–Olkin (KMO) = 0.914, Bartlett's Test of Sphericity was significant, χ2 = 2378.9, p < 0.001, therefore principal component analysis (PCA) was run. SPSS R-Menu v2.0 was used for determining the criteria for retaining factors in EFA (Courtney and Gordon 2013). Velicer’s Squared Minimum Average Partial test suggested a 3 factor solution and Comparative Data test (Ruscio and Roche, 2012) suggested that moving from 2 factor to 3 factor solution provided a statistically significant improvement to model fit (p < 0,001), whereas moving from a 3 factor to 4 factor solution did not provide a significant improvement in model fit (p = .770). In summary, test results converge on the suitability of retaining a 3 factor solution. These three factors explain 58.9% of the variance, which is similar to results obtained by Baka (2017).

Promax rotation with Kaiser normalisation was applied to the PCA and a cut-off value of 0.40 was used for analysis of factor loadings (Hair et al., 2006). Pattern coefficients are presented in Table. Pattern coefficients include a control for the contribution of other factors on a particular item. Analysis suggests that items load on factors differently than in the study by Baka (2017).

The first, main factor, which explains 46.64% of variance consists of general teaching effectiveness, understood as both adjustment of teaching to individual needs, as well as maintenance of high general effectiveness and motivation. Second factor, 6.69% of variance relates to maintenance of relationships and mutual expectations with teachers, administration and parents, Third factor, 5.57% of variance, relates to maintenance of discipline.

The motivational and adjustment elements of teaching were separate factors in the study by Baka (2017). Possibly the reason for the discrepancy of findings might be due to the specific sample of the current study. This study included relatively experienced mathematics teachers. That is, these are teachers willing to invest some of their own resources for skill training. It is likely that for them the motivational aspects of education are indistinguishable from adjustment of the education program or use of various methods. Perhaps in a more general sample there is a discrepancy between teachers who focus only on motivation but cannot adjust the program or vice versa.

In summary, current study utilizes a 3 factor solution, but the factors are: a) General Teaching Effectiveness (Items: 5,1,18,23,11,10,17,16,21,8,15,4), b) Relationships Maintenance (Items:22,3,7,24,13), c) Discipline (6,9,2,14,19). 2 items were dropped (20, 12), as they did not strongly load on any of the factors.

| *Pattern Component Matrix^a^* | | | |
| --- | --- | --- | --- |
|  | Pattern Coefficents | | |
|  | 1 | 2 | 3 |
| N5... zorganizować pracę tak, aby metoda nauczania i wykonywane przez uczniów zadania były dopasowane do indywidualnych potrzeb. / “How certain are you that you can organize schoolwork to adapt instruction and assignments to individual needs?” | ,887 |  |  |
| N1... wyjaśnić najważniejsze zagadnienia omawianego tematu w taki sposób, aby zrozumieli je nawet uczniowie ze słabymi osiągnięciami w nauce. / “How certain are you that you can explain central themes in your subjects so that even the low-achieving students understand?” | ,816 |  |  |
| N18... dostosować nauczanie do możliwości mniej zdolnych uczniów, nie zaniedbując jednak potrzeb pozostałych uczniów. / “How certain are you that you can adapt instruction to the needs of low-ability students while you also attend to the needs of other students in class?” | ,805 |  |  |
| N23... organizować pracę na lekcji w taki sposób, aby zarówno uczniowie bardziej, jak i mniej zdolni wykonywali zadania dopasowane do ich możliwości. / “How certain are you that you can organize classroom work so that both low- and high-ability students work with tasks that are adapted to their abilities?” | ,804 |  |  |
| N11... stawiać realistyczne wymagania uczniom, nawet w klasie zróżnicowanej pod względem zdolności uczniów./ “How certain are you that you can provide realistic challenge for all students even in mixed ability classes?” | ,755 |  |  |
| N10... wzbudzić chęć uczenia się nawet wśród uczniów z najsłabszymi osiągnięciami./ “How certain are you that you can wake the desire to learn even among the lowest achieving students? | ,739 |  |  |
| N17... efektywnie nauczać bez względu na skład klasy, czy zróżnicowanie uczniów pod względem wieku. / “How certain are you that you can manage instruction regardless of how it is organized (group composition, mixed age groups, etc.)?” | ,712 |  |  |
| N16... przedstawić omawiany temat tak, aby większość uczniów zrozumiała podstawowe zagadnienia. / “How certain are you that you can explain subject matter so that most students understand the basic principles?” | ,676 |  |  |
| N21... zmotywować uczniów przejawiających małe zainteresowanie nauką. / “How certain are you that you can motivate students who show low interest in schoolwork?” | ,631 |  |  |
| N8... dobrze kierować i nauczać wszystkich uczniów, niezależnie od poziomu ich zdolności. / “How certain are you that you can provide good guidance and instruction to all students regardless of their level of ability?” | ,625 |  |  |
| N15... sprawić, aby uczniowie „dawali z siebie wszystko”, nawet gdy pracują nad trudnymi problemami. / “How certain are you that you can get students to do their best even when working with difficult problems?” | ,563 |  | ,446 |
| N4... skutecznie stosować metody nauczania obowiązujące w szkole. / “How certain are you that you can successfully use any instructional method that the school decides to use?” | ,439 |  |  |
| N20... poradzić sobie z nauczaniem, nawet jeśli zmieniony został program nauczania. / “How certain are you that you can manage instruction even if the curriculum is changed?” |  |  |  |
| N12... odpowiadać na pytania uczniów tak, aby zrozumieli oni nawet skomplikowane zagadnienia. / “How certain are you that you can answer students’ questions so that they understand difficult problems?” |  |  |  |
| N22... efektywnie i konstruktywnie współpracować z innymi nauczycielami, na przykład nauczającymi tego samego przedmiotu. / “How certain are you that you can cooperate effectively and constructively with other teachers, for example, in teaching teams?” |  | ,715 |  |
| N3... dobrze współpracować z większością rodziców. / “How certain are you that you can cooperate well with most parents?” |  | ,673 |  |
| N7... znaleźć odpowiednie rozwiązanie konfliktów występujących między nauczycielami. / “How certain are you that you can find adequate solutions to conflicts of interest with other teachers?” |  | ,667 |  |
| N24... dobrze nauczać, nawet jeśli oczekiwano by od Ciebie stosowania takich metod nauczania, które Ci nie odpowiadają. / “How certain are you that you can teach well  even if you are told to use instructional methods that would not be your choice?” |  | ,635 |  |
| N13... konstruktywnie współpracować z rodzicami uczniów sprawiających problemy wychowawcze. / “How certain are you that you can collaborate constructively with parents of students with behavioral problems?” |  | ,555 |  |
| N6... utrzymać dyscyplinę na lekcji lub w grupie uczniów. / “How certain are you that you can maintain discipline in any school class or group of students?” |  |  | ,899 |
| N9... zapanować nawet nad najbardziej agresywnymi uczniami. / “How  certain are you that you can control even the most aggressive students?” |  |  | ,808 |
| N2... sprawić, aby wszyscy uczniowie w klasie intensywnie pracowali w szkole i wykonywali zadania domowe. / “How certain are you that you can get all students in class to work hard with their schoolwork?” |  |  | ,656 |
| N14... sprawić, aby uczniowie przejawiający problemy wychowawcze przestrzegali zasad obowiązujących w klasie. / “How certain are you that you can get students with behavioral problems to follow classroom rules?” |  |  | ,649 |
| N19... sprawić, aby uczniowie uprzejmie i z szacunkiem odnosili się do nauczycieli. / “How certain are you that you can get all students to behave politely and respect the teachers?” |  |  | ,506 |
| Extraction Method: Principal Component Analysis. Rotation Method: Promax with Kaiser Normalization. | | | |
| a. Rotation converged in 8 iterations. | | | |
